# Evaluating the Wasfaty E-Prescribing Platform Against Best Practices for Computerized Provider Order Entry

**DOI:** 10.3390/healthcare13080946

**Published:** 2025-04-20

**Authors:** Saba Alkathiri, Razan Alothman, Sondus Ata, Yazed Alruthia

**Affiliations:** 1 Pharmacoeconomics Research Unit, College of Pharmacy, King Saud University, Riyadh 11451, Saudi Arabia; 441200466@student.ksu.edu.sa (S.A.); 441200365@student.ksu.edu.sa (R.A.); 2 Pharmacy Services Department, King Saud University Medical City, Riyadh 11451, Saudi Arabia; sata@ksu.edu.sa; 3 Department of Clinical Pharmacy, College of Pharmacy, King Saud University, Riyadh 11451, Saudi Arabia

**Keywords:** adverse drug reactions, e-prescribing, outpatient, patient safety, electronic medical records, Saudi Arabia

## Abstract

**Background:** Saudi Arabia is undertaking a comprehensive reform of its healthcare system to improve the efficiency and accessibility of public healthcare services. A key aspect of this initiative is outsourcing outpatient pharmacy services within the public health sector to retail pharmacies through an electronic prescribing platform known as Wasfaty. The National Unified Procurement Company (NUPCO) manages this platform to ensure spending efficiency and patient accessibility to essential medications. However, there has been a lack of research evaluating the adherence of the Wasfaty e-prescribing platform to established best practices for Computerized Provider Order Entry (CPOE), which are commonly used to assess the performance of various ambulatory e-prescribing systems globally. **Objective:** This study aimed to assess the level of adherence of Wasfaty to best practices for CPOE. **Methods:** This descriptive cross-sectional single-center study reviewed filled prescriptions through Wasfaty from May 2022 to December 2023. A list of 60 functional features, including but not limited to patient identification and data access, medication selection, alerts, patient education, data transmission and storage, monitoring and renewals, transparency and accountability, and feedback, was utilized to evaluate adherence. The adherence level was categorized into four groups: fully implemented, partially implemented, not implemented, and not applicable. Two pharmacy interns, a clinical pharmacist, and a researcher, reviewed the prescriptions to determine the platform’s adherence to these 60 CPOE features. **Results:** From May 2022 to December 2023, a total of 1965 prescriptions were filled in retail pharmacies for out-of-stock medications for 1367 patients. These prescriptions included medications for various areas, with the following distribution: gastroenterology (44.10%), cardiology (18.14%), anti-infectives (2.42%), urology (8.85%), dermatology (3.6%), hematology (0.29%), muscle relaxants (0.8%), neurology (19.17%), pulmonology (1.46%), and other categories (1.23%). Of the 60 functional characteristics a CPOE platform should include, only 19 (31.66%) were fully implemented, while 10 (16.66%) were partially implemented. **Conclusions:** The Wasfaty platform is deficient in several key functional features necessary for e-prescribing, which are essential for ensuring patient safety and enhancing the satisfaction of both prescribers and patients. This study underscores the importance of improving the Wasfaty platform to reduce the risk of adverse drug events.

## 1. Introduction

The implementation of Computerized Provider Order Entry (CPOE) has integrated outpatient pharmacy orders with electronic health records (EMRs), significantly enhancing medication prescribing through a structured framework supported by Clinical Decision Support Systems (CDSSs) [1]. EMRs present numerous advantages in healthcare technology that can significantly enhance patient care. EMRs facilitate improvements in the quality and reliability of patient information by providing a centralized and easily accessible digital platform for healthcare providers [2]. The integration of CPOE with EMRs reduces administrative costs associated with paper records and allows for identifying clinical areas that require attention through data analysis [2,3]. CPOE optimizes prescriptions tailored to patient characteristics and diminishes the incidence of medication errors [1]. Key requirements for CPOE include access to patient information, medication lists, laboratory results, medical histories, and compliance with cybersecurity standards to protect privacy [4,5]. Integrating Clinical Decision Support Systems (CDSSs) into EMRs represents a significant advancement in healthcare technology, as it allows for the seamless amalgamation of medication information with comprehensive patient data [6]. This integration is crucial for providing clinicians with timely and pertinent alerts concerning potential drug–drug interactions and contraindications associated with specific medications, including those related to food allergies and other critical health situations [1,4].

Moreover, CPOE addresses issues related to handwritten prescriptions and medication theft while providing automatic updates regarding formularies and insurance coverage, which enhances communication and workflow among healthcare providers. The benefits encompass quicker prescription refills, reduced wait times, and increased convenience for patients, leading to substantial cost savings, as evidenced by several studies [6,7,8]. One study indicated that the incremental cost-effectiveness ratio for CPOE in preventing additional medication errors was EUR 3.54, highlighting its cost-effectiveness [8].

The ambulatory e-prescription systems have significantly improved patient safety and satisfaction globally [9]. Numerous research studies have documented the benefits of e-prescribing, including reductions in adverse drug events, prescription errors, and interactions between drugs and food or other medications [10,11]. Additionally, e-prescriptions enhance efficiency by minimizing the time required to exchange and retrieve medical data [9,11]. Countries such as Turkey, Australia, Spain, Japan, Sweden, Denmark, and the United Kingdom have successfully implemented national e-prescription systems, reporting favorable outcomes in patient and prescriber satisfaction and clinical results [12].

A recent comprehensive study explored various perspectives regarding the effects of healthcare privatization, including the privatization of outpatient pharmacy services. Among the various studies analyzed, four presented findings that supported the notion of privatization, with two of these studies conducted explicitly in Saudi Arabia [13]. Conversely, six studies opposed privatization, citing potential drawbacks, while one maintained a neutral stance [13]. The implications of healthcare privatization are complex and highly context-dependent, leading to divergent opinions about its impact on critical areas such as the quality, equity, and accessibility of healthcare services [14]. Additionally, discussions around its cost-effectiveness remain multifaceted, with advocates often emphasizing potential efficiency gains, while critics warn of exacerbated inequalities and reduced service quality for vulnerable populations [14]. Therefore, healthcare policy researchers advocate for aligning healthcare privatization policies with the principles of patient-centered care within Arabic culture to avoid the negative consequences of healthcare privatization [15].

In Saudi Arabia, the initiative aiming for 70% digitization of patient services by 2030 aligns with broader economic reforms, enhancing healthcare accessibility. The Wasfaty e-prescribing platform, developed in Saudi Arabia, enhances healthcare efficiency by connecting hospitals and pharmacies. It allows patients to conveniently access their prescribed medications from nearby pharmacies. A key feature of Wasfaty is its real-time communication between healthcare providers and pharmacies, which ensures accurate and prompt prescription filling and minimizes errors linked to traditional paper prescriptions. It employs stringent safety measures, including secure access and encryption, to protect patient information [16]. Evaluations of the Wasfaty program have reported improved patient experiences and operational efficiency, although challenges persist, such as increased workload for pharmacists and patient dissatisfaction stemming from medication shortages [17,18]. NUPCO initiated and oversees the Wasfaty program, which functions as a centralized procurement entity for all public healthcare organizations in Saudi Arabia, facilitating the acquisition of pharmaceuticals and medical devices. Public healthcare institutions must procure medicines and medical devices through NUPCO and may only outsource their outpatient services via the Wasfaty program.

Although research studies have shown controversial results regarding patient experience and medication availability with Wasfaty program [19], no study so far examined the adherence of this e-prescribing platform to the best practices for Computerized Provider Order Entry (CPOE). These practices include the ability to access basic demographic data about the patients, medication history, support in choosing the appropriate prescription medication, patient education tools, data transmission and storage, and protection of patient information [4,20,21]. So far, the adherence of the Wasfaty program to the best practices in ambulatory e-prescribing platforms has not been examined. Therefore, this study aimed to explore the adherence of this national outpatient e-prescribing platform to the best practices for CPOE.

## 2. Methods

### 2.1. Study Design and Data Collection

This study utilized a retrospective cohort design to examine outpatient prescriptions processed through the Wasfaty program at King Khalid University Hospital, a prominent university-affiliated tertiary care facility with more than 1200 staffed beds in Riyadh, Saudi Arabia. While the majority of outpatient prescriptions are typically filled by the hospital’s own pharmacy, there are instances where certain medications are unavailable—either due to stock shortages or because specific dosage formulations requested by prescribers are not offered by the institution. In such cases, these prescriptions are filled through the Wasfaty program, which operates a distinct e-prescribing portal. This portal is designed to be accessed by authorized pharmacists and prescribers, ensuring that requests are limited to items that cannot be obtained within the hospital. The Wasfaty program is managed by NUPCO, a centralized procurement company that serves as the primary resource for all public healthcare organizations in Saudi Arabia in terms of acquiring pharmaceuticals and medical devices. The scope of this study encompassed data retrieval covering the period from May 2022 to September 2023.

### 2.2. An Overview of E-Prescribing Systems in Different Countries

Globally, e-prescribing systems share common goals: to streamline the prescription process and enhance patient safety. However, differences in their structure exist. Some systems are centralized, storing data in national databases, while others are decentralized, allowing individual facilities to maintain data. The Wasfaty e-prescribing platform is decentralized, and institutional data are mostly unlinked to this platform [18]. On the other hand, the UK’s Electronic Prescription Service (EPS) is an integral part of the National Health Service (NHS). It allows prescribers to send electronic prescriptions directly to a patient’s chosen pharmacy, handling about 1.5 million prescriptions daily. The EPS emphasizes patient consent and utilizes the NHS Spine—a secure central system for healthcare information exchange—improving safety and efficiency. Patient safety is crucial within the EPS, incorporating CDSS to alert prescribers about potential drug interactions and allergies. Additionally, unique identifiers for prescriptions and smartcard authentication for providers enhance security, and regular audits promote a safer prescribing environment compared with a decentralized system, such as the Wasfaty e-prescribing platform [12,22]. Spain has similarly implemented an e-prescribing system to improve patient safety and streamline communication between healthcare providers and pharmacies [12]. Furthermore, Turkey’s e-prescribing system, the e-Prescription System (e-Reçete), was established to streamline the prescription process and enhance patient safety. This e-prescription system is integrated with the national health information system, granting prescribers access to patients’ medication histories, including previous prescriptions and potential drug interactions [5,11,12].

### 2.3. E-Prescribing Functional Characteristics Used to Assess the Wasfaty Program Compliance

To evaluate the compliance of the Wasfaty program with best practices in Computerized Provider Order Entry (CPOE), the research focused on 10 specific domains encompassing 60 essential functional characteristics deemed crucial for any e-prescribing platform aimed at safeguarding patient safety [20,23]. These functional characteristics were developed by a panel of experts from different professional domains related to electronic prescribing (i.e., medicine, nursing, pharmacy, managed care, consumer advocacy, healthcare oversight, healthcare economics, healthcare safety and quality, and medical informatics) with a clear objective to help decision-makers and policymakers select the best ambulatory e-prescribing systems that ensure optimal patient outcomes [4,21,23]. The 60 functional characteristics covered the following domains:

1. Patient identification and data access—ensuring that relevant demographic information is readily available.
2. Current medications and medication history—enabling prescribers to access a comprehensive list of a patient’s current and past medications.
3. Medication selection—assisting in the selection of appropriate medications based on entered diagnoses and the patient’s medical background.
4. Alerts and communications—providing alerts that inform prescribers about potential contraindications and guiding them towards optimal medication choices based on individual patient data.
5. Patient education—supplying necessary information to patients on the proper administration of prescribed medications.
6. Data transmission and storage—allowing prescribers to directly transmit prescriptions to the patient’s preferred pharmacy.
7. Monitoring and renewals—ensuring that the system notifies prescribers if a prescription or its refill has not been dispensed.
8. Transparency and accountability—disclosing clear and transparent procedures regarding how clinical decision support rules are formulated.
9. Prescriber-level feedback—providing prescribers with insights to review their own prescribing patterns.
10. Security and confidentiality—supporting adherence to regulations designed to protect patient privacy and maintain the confidentiality of health information.

To determine adherence to these 60 functional characteristics, a group comprising two pharmacy interns, a clinical pharmacist, and a researcher meticulously reviewed the outpatient prescriptions. They employed a three-tiered system for assessing implementation levels: fully implemented, partially implemented, and not implemented. Furthermore, assessors had the option to classify certain functional characteristics as “not applicable” if they did not pertain to the context of the Saudi public healthcare sector, such as in instances of sponsorship disclosure. Prior to the review process, a thorough examination of the COPE’s functional characteristics was conducted, followed by a meeting to ensure that each team member fully understood all listed characteristics of the CPOE. Throughout the study, the reviewers held two meetings to collaboratively assess the functional characteristics and verify the adherence level for each one evaluated. Disagreements among team members regarding compliance with specific functional characteristics were only considered valid if they could provide concrete evidence from the reviewed prescriptions to back up their arguments. In the absence of such evidence, the team would resolve the rating through a unanimous consensus, ensuring everyone’s opinion was acknowledged while maintaining clarity and consistency in the decision-making process. 

### 2.4. Statistical Analysis

The data were sorted and coded prior to the analysis, and they did not include personal identifiers such as names, IDs, or addresses, nor were they retrieved from the Wasfaty system. Descriptive statistics using mean, median, standard deviation, frequencies, and percentages were used to present the patient characteristics, and prescription medication classes using the World Health Organization anatomical therapeutic chemical classification [24]. A Student *t*-test was used to compare the mean age and weight across genders. All statistical analyses were conducted using SAS^®^ version 9.4 (SAS^®^ institute, Cary, NC, USA).

## 3. Results

**Figure 1:**
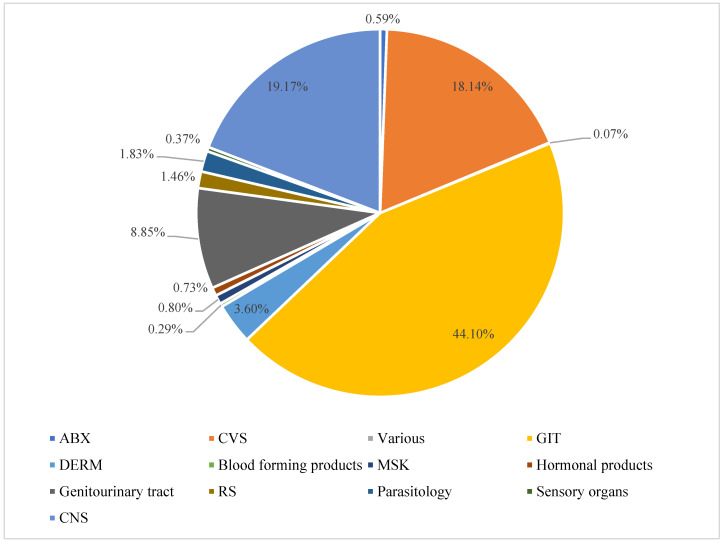
Medication classes distributions. Abbreviations: ABX: Antibiotics, CVS: Cardiovascular system products, Various: Various products, GIT: Gastrointestinal tract products, DERM: Dermatological products, MSK: Musculoskeletal system products, Genitourinary tract: Genitourinary tract products, RS: Respiratory system products, Parasitology: Parasitology products, Sensory organs: Sensory organs products, CNS: Central nervous system products.

From 2 May 2022 to 30 September 2023, 1966 prescription medications were dispensed to 1367 patients through the Wasfaty program. These prescription medications were prescribed through the Wasfaty program either because certain prescription medications were out of stock or due to the unavailability of specific dosage formulations or strengths. About 55% of the patients were females; however, the mean age of the male patients was slightly higher than that of their female counterparts (55.65 versus 53 years). Male patients also had a higher mean weight than their female counterparts (75.45 kg vs. 73.39 kg, *p*-value = 0.0042), as shown in Table 1. The mean weights for different age groups and across genders are shown in Table 2. Using the WHO anatomical therapeutic chemical (ATC) classification, gastrointestinal tract drugs, such as antacids, anticholinergic agents, and proton pump inhibitors, represented approximately 44% of the reviewed prescriptions. Central nervous system (e.g., antiepileptics, analgesics, antiemetics, and antiparkinsonian agents, etc.) and cardiovascular system drugs (e.g., cholesterol-lowering medications, anticoagulants, antiplatelets, beta-blockers, etc.) represented 19.17% and 18.14% of the prescriptions, respectively. Genitourinary tract products (e.g., alpha-blockers, urinary antispasmodics, impotence agents) and dermatological products (e.g., topical antibiotics, topical antivirals, topical antifungals, and topical steroids) represented 8.85% and 3.60% of the prescriptions, respectively. Other classes of prescription medications represented less than 2% of the reviewed prescriptions, as shown in Figure 1.

### 3.1. Patient Identification and Data Access

**Figure 2:**
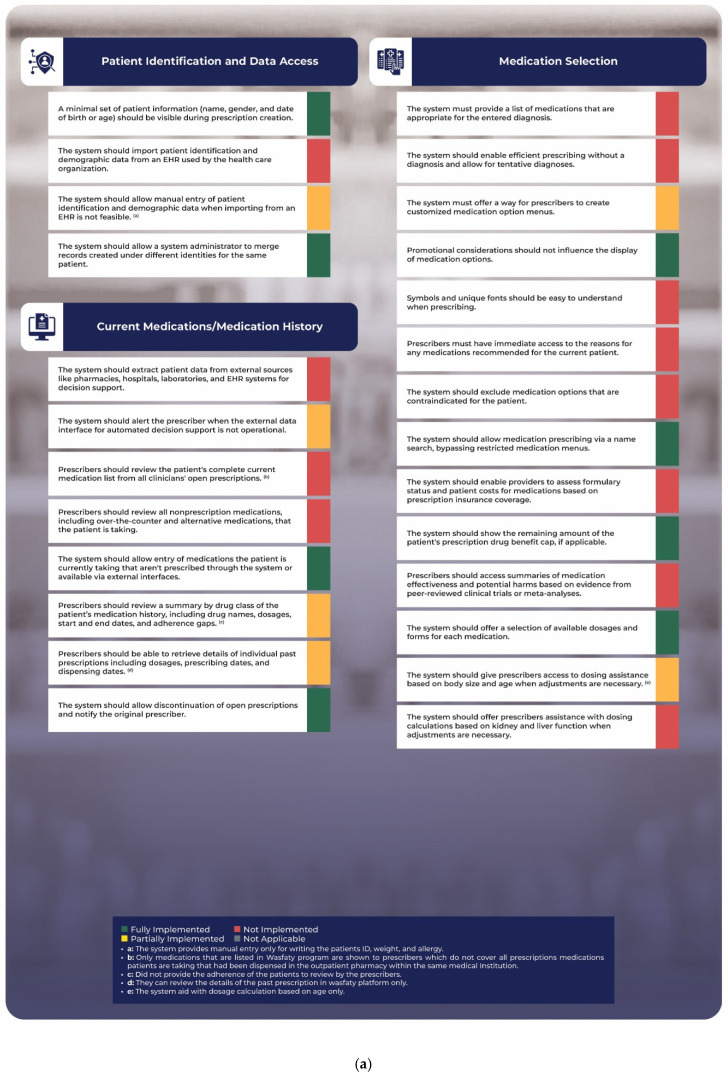

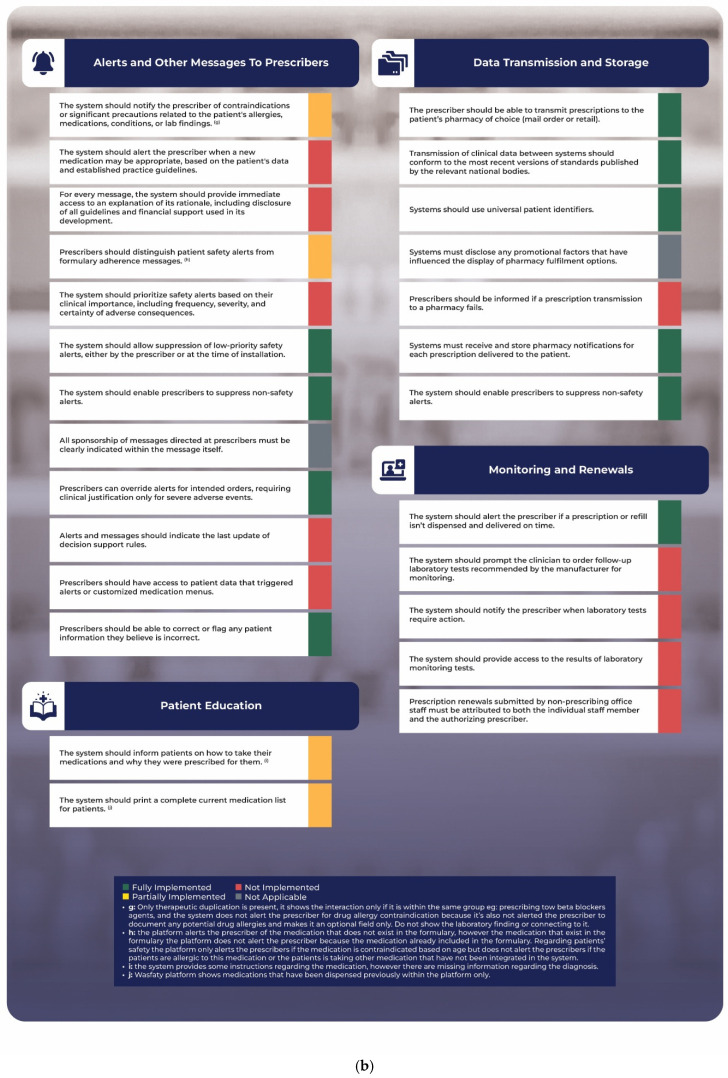

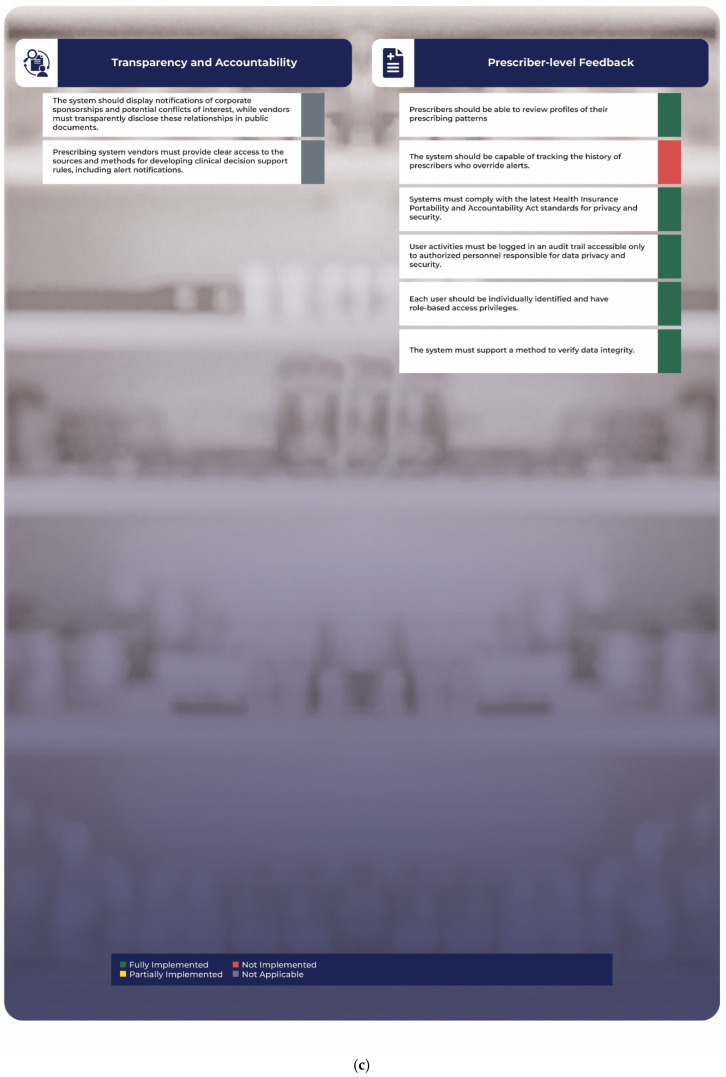
Functional characteristics that should be available in any commercial ambulatory electronic prescribing system. (**a**) Patient identification and data access, current medications/medication history, and medication selection. (**b**) Alerts and other messages to prescribers, patient education, data transmission and storage, and monitoring and renewals. (**c**) Transparency and accountability, and prescriber-level-feedback.

Two functional characteristics in the domain of patient identification and data sharing were successfully implemented. However, access to demographic and patient identification data from the electronic medical record (EMR) was not fully realized. Conversely, the system allows for the partial manual entry of certain patient identification and demographic information—such as patient ID, weight, and allergies—when it is not integrated with the EMR, as illustrated in Figure 2a.

### 3.2. Current Medications/Medication History

The Wasfaty platform does not permit the extraction of patient data from external sources such as community pharmacies, electronic medical records (EMRs), hospitals, and laboratories to facilitate decision support. Additionally, the Wasfaty platform only notifies prescribers when essential information regarding the appropriateness of prescriptions is missing, such as age, but it does not warn them about critical information regarding potential drug–drug interactions or therapeutic duplications, as it lacks integration with other external sources like EMRs. Prescribers can access only active prescriptions within the Wasfaty platform and are unable to review a patient’s complete list of medications stored in their EMRs, despite both platforms functioning within the same hospital. That said, the Wasfaty platform does allow for the manual entry of medications that patients may be taking but are not displayed within the system. Although prescribers can view the start and end dates of all prescriptions on the Wasfaty platform, they are unable to identify any gaps in adherence since the system does not encompass the patient’s comprehensive medication history. Furthermore, prescribers can only retrieve data on prescriptions issued through the Wasfaty platform and cannot access information on other prescriptions dispensed within the same hospital. On a positive note, the Wasfaty platform does provide prescribers with the capability to discontinue any active prescriptions within the system (Figure 2a).

### 3.3. Medication Selection

There are 14 functional characteristics in the medication selection domain, and the Wasfaty platform fully implemented only 4 of them, as shown in Figure 2a. The Wasfaty platform does not suggest a list of medications appropriate for the entered diagnosis. Moreover, the Wasfaty platform does not allow for prescribing with a definitive diagnosis. Although the Wasfaty platform enables the entry of proper medications based on age and the absence of significant drug–drug interactions for patients, this only applies to drugs that are listed within the Wasfaty platform and does not allow prescribers to create a customized menu of medications for each patient based on their medical profile. The Wasfaty platform does not ask the prescriber to clarify any included symbols in the prescriber notes.

Additionally, the Wasfaty platform does not provide the prescribers with any rationale for suggested medications. Moreover, it does not exclude medication options deemed inappropriate for patients. Furthermore, the system does not enable the prescribers to check the formulary status of medications or costs patients may incur based on their insurance coverage since it is currently designed for public institutions only. In addition, the system does not provide access to essential clinical information about the entered medical diagnosis or prescribed medications. Although the Wasfaty platform alerts prescribers if an inappropriate drug or dosage was entered based on the age of the patient, it does not take the weight and renal or liver functions into account. Additionally, it offers no assistance with dosing calculations based on weight and renal or liver functions.

### 3.4. Alerts and Other Messages to Prescribers

A review of 12 functional characteristics in the alerts and messages directed at prescribers revealed that only four were fully implemented, two were partially implemented, one was deemed inapplicable, and five were not implemented, as illustrated in Figure 2b. For instance, the system alerts prescribers to potential therapeutic duplication based on prescriptions entered on the same platform; however, it fails to notify them about potential drug allergies or assess the appropriateness of prescriptions in relation to the patient’s medical profile. Additionally, there is no immediate explanation or clarification provided for the messages that appear on the platform.

While the system does inform prescribers when they prescribe a medication outside of the formulary, it does not address the appropriateness of those medications in the context of the patient’s medical history. The system also lacks a prioritization of safety alerts based on their clinical significance. Furthermore, it does not provide information regarding updates to decision support rules, nor does it give prescribers access to the data that inform alerts about medication appropriateness. Lastly, the system prohibits sponsored messages directed at prescribers.

### 3.5. Patient Education

In the field of patient education, the Wasfaty platform primarily focuses on providing printed instructions for patients about the correct use of their prescribed medications. However, a notable limitation of this platform is that the instructions it delivers are generic and do not consider the individual clinical conditions or unique healthcare needs of the patients. This lack of customization means that patients may not receive guidance that fully addresses their specific circumstances, potentially impacting their understanding and adherence to medication regimens.

### 3.6. Data Transmission and Storage

The data transmission and storage domain includes seven unique functional characteristics, of which five have been successfully realized and illustrated in Figure 2b. However, the system lacks a crucial feature: it does not notify prescribers when a prescription transmission to a pharmacy fails. This shortcoming creates significant challenges for patients, who often have to return to their healthcare providers to report that the selected pharmacy has not received their prescriptions. Such a scenario complicates the patient experience by introducing an unnecessary additional step and raises serious concerns about the overall efficiency and reliability of the prescription process. This deficiency can harm patient care and medication adherence, potentially leading to missed doses or delays in treatment.

### 3.7. Monitoring and Renewals

The system has only partially implemented one of the five essential functional characteristics related to monitoring and prescription renewals (Figure 2b). Specifically, it cannot actively prompt clinicians to order follow-up laboratory or imaging tests when necessary, leading to delays in patient care. Moreover, the system does not alert clinicians regarding laboratory test results that require immediate attention, potentially resulting in critical information being overlooked. In addition to these shortcomings, there is a significant barrier to effective communication among healthcare providers. Pharmacists and prescribers cannot access laboratory test results, which limits their capacity to make informed decisions regarding patient treatment and medication management. Another issue arises when non-prescribing medical staff, such as nurses or pharmacists, submit a prescription renewal request. When authorization from a prescribing physician is required, the system fails to indicate who initiated the renewal request. Instead, it only presents the details associated with the physician authorizing the request, which can create confusion and impede the workflow among the healthcare team. This lack of transparency can complicate the prescription renewal process and potentially affect patient safety and care continuity.

### 3.8. Transparency and Accountability 

The transparency and accountability domain comprises two essential functional characteristics, neither of which are presently being fulfilled. Specifically, the system should proactively identify and manage potential conflicts of interest related to corporate sponsorships. Additionally, it should disclose any conflicts associated with its creation by a for-profit corporation under government ownership. This lack of transparency raises concerns about the integrity of the platform. Furthermore, as demonstrated in Figure 2c, the platform fails to provide users with access to vital sources for developing clinical decision support rules or alert notifications, thereby limiting its effectiveness in supporting healthcare professionals.

### 3.9. Prescriber-Level Feedback

Although five of the six functional characteristics in the prescriber-level feedback domain were fully implemented, the system cannot track the history of the prescribers who override alerts, as shown in Figure 2c.

## 4. Discussion

A Computerized Physician Order Entry (CPOE) platform must meet specific criteria to facilitate efficient and accurate medication prescribing [20]. During our comprehensive assessment of the Wasfaty platform, we identified a total of 60 critical functional characteristics that are essential for any effective CPOE system [4,20]. Out of these, only 19 features, representing 31.66%, were found to be fully implemented and operational. In addition, 10 features, or 16.66%, were only partially implemented, meaning they meet some but not all necessary requirements. Furthermore, we discovered that several key requirements, as outlined in established good practice guidelines for CPOE systems, were either missing entirely or needed significant enhancements to align with best practices [4,25]. This evaluation underscores the necessity for improvements to ensure that the Wasfaty platform can provide the optimal functionality required for safe and effective medication management.

The absence of contextually relevant CDSS alerts, which is one of the key limitations of the Wasfaty e-prescribing platform, can lead to inappropriate prescribing practices, posing risks to patient safety and treatment efficacy [26]. For instance, a study that examined the value of CDSSs in helping physicians in developing customized stroke treatments in Korea for stroke thrombolysis therapy among patients with acute ischemic stroke demonstrated the value of these systems [27]. In another pilot study that examined the value of clinical decision support tools to screen health records for contraindications to stroke thrombolysis, the utilization of CDSSs notably reduced the amount of time needed for healthcare professionals to review patient records, enhancing efficiency in clinical workflows [28]. Moreover, this approach led to a significant increase in both the quantity and quality of contraindications detected for thrombolysis therapy, ultimately contributing to better patient outcomes and more informed decision-making in critical care situations, as demonstrated by multiple studies including a recently published study that examined the value of integrating CDSSs within CPOE for improving venous thromboembolism risk assessment using real-world data [29].

To effectively diagnose patients within the Wasfaty system, it is essential to input diagnoses using the International Classification of Diseases, Tenth Revision (ICD-10) codes [30]. However, a significant limitation of the platform is its failure to accommodate common off-label indications for various treatments. This oversight can result in the rejection of certain diagnoses, which restricts clinicians’ ability to provide comprehensive care. Furthermore, the system does not integrate critical laboratory and imaging results, which are vital for physicians to draw accurate and informed conclusions about a patient’s condition. Moreover, a recent study focused on the efficacy of combining CPOE with CDSSs in the context of radiological services presents compelling evidence [31]. This integration has the potential to streamline the ordering process, significantly reduce healthcare costs, and address persistent challenges associated with medical imaging. By enhancing the decision-making process for physicians, such collaboration not only improves the accuracy of diagnoses but also elevates the overall quality of patient care [31].

Although the Wasfaty platform is supposed to improve the quality of care and patient safety as the kingdom is keen to increase the private sector participation in healthcare provision, this private CPOE platform failed to meet numerous standards necessary to ensure patient safety and improve the patient experience. One significant limitation is the lack of integration between the Wasfaty platform and the EMRs. By incorporating EMRs into the Wasfaty platform, healthcare institutions can ensure that patient data are consistently available across different facilities. This minimizes the risk of duplicated medical records and reduces the likelihood of medication errors—issues crucial for patient safety and optimal care delivery [32]. Moreover, the integration of over-the-counter (OTC) medications into patients’ EMRs presents a nuanced and multifaceted challenge that has the potential to enhance clinical outcomes and improve overall patient care [3,32]. Currently, the documentation practices concerning patient allergies within EMRs are often inadequate. Many allergy records do not include detailed information regarding specific reactions, while relevant details about the nature and severity of those allergies are frequently incomplete. A significant number of recorded reactions are often designated as “unknown”, which introduces a substantial safety risk, particularly when a patient has a coded allergy that could be easily overlooked by healthcare providers during treatment.

Additionally, research conducted by Márquez-Contreras et al. validated the use of electronic prescription to measure patient adherence to their prescribed medications using the medication event monitoring system (MEMS), and e-prescription showed both high sensitivity and specificity among patients with hypertension [33]. By tracking patient medication usage and providing insightful data, e-prescription, if equipped with adherence assessment tools, can offer healthcare professionals valuable perspectives on the effectiveness of various medical treatments, thus facilitating more informed decision-making that can ultimately benefit patient health and wellbeing. Unfortunately, the Wasfaty platform cannot provide accurate estimates of patient adherence to medications since it is not integrated with the EMRs.

### Limitations and Strengths

Although this is the first study, to the best of our knowledge, that examined the compliance of the Wasfaty e-prescribing platform with the essential functional characteristics that must exist in electronic prescription platform in Saudi Arabia and highlighted numerous shortcomings that have not been highlighted before, multiple limitations of this study must be acknowledged. Despite the fact that the platform is almost identical in different public healthcare institutions, this is a single-center study, which limits the generalizability of the findings. Moreover, the panel who reviewed and evaluated the Wasfaty platform were mostly pharmacists and did not include other relevant professions, such as medicine, nursing, and medical informatics. Furthermore, there might be technical or logistical reasons behind the failure of the Wasfaty program to adhere to the review functional characteristics for CPOE that the investigators of this study might not privy to.

## 5. Conclusions

While CPOE systems have significantly enhanced healthcare delivery and patient satisfaction, their adherence to crucial standards for safe and effective prescribing varies. The Wasfaty platform represents the first private CPOE system launched for public healthcare institutions in Saudi Arabia, coinciding with the kingdom’s comprehensive transformation of its healthcare system to improve the quality of services and enhance spending efficiency. However, numerous issues related to the adherence of this CPOE platform to best practices in electronic prescribing could lead to diminished patient experience and unfavorable clinical outcomes, as indicated by the results of this study. Consequently, the findings should inform policymakers and decision-makers in Saudi Arabia’s public health sector about addressing the identified gaps to ensure patient safety and enhance patient experience. Integrating the Wasfaty platform with the EMRs should be the first step in addressing many of these ambulatory e-prescribing platform’s identified limitations. In addition, the Wasfaty platform should undergo review by experts with relevant expertise, such as in medicine, pharmacy, nursing, patient safety, health economics, and medical informatics, among others. This review should take place after addressing the identified gaps to ensure the platform aligns with the best practices in CPOE aimed at enhancing patient outcomes and the experience of prescribers.

## Figures and Tables

**Table 1:** Patient baseline characteristics.

Variable	Male (n = 612)	Female (n = 755)	*p*-Value	All Patients (n = 1367)
Age (years)	55.65 ± 20.48	52.99 ± 19.44	0.0011	54.19 ± 19.95
Weight (kg)	75.45 ± 20.43	73.39 ± 18.80	0.0042	74.32 ± 19.57

**Table 2:** Age groups and weight across gender.

Age in Years	Percent (%)	Number of Males	Weight for Male (Mean ± SD)	Number of Females	Weight for Female (Mean ± SD)
1 to <5	1.46	11	25.55 ± 30.3	9	15.00 ± 6.40
5 to ≤10	2.27	14	23.79 ± 5.69	17	28.21 ± 12.90
>10 to ≤15	2.71	21	39.95 ± 13.60	16	49.88 ± 17.11
>15 to ≤20	2.27	12	58.83 ± 28.22	19	58.37 ± 17.89
>20 to ≤25	3.07	14	69.29 ± 29.03	28	64.75 ± 18.92
>25 to ≤30	6.22	29	77.93 ± 20.72	56	68.57 ± 13.95
>30 to ≤35	9.14	49	90.80 ± 18.64	76	77,53 ± 18.85
>35 to ≤40	15.22	84	80.89 ± 16.97	124	79.68 ± 16.68
>40 to ≤45	27.36	163	80.73 ± 12.51	211	78.09 ± 14.14
>45	30.29	215	76.20 ± 11.88	199	75.33 ± 13.62

## Data Availability

The data are available upon reasonable request from the corresponding author.
